# Nutritional composition and health benefits of peas—a bibliometric research

**DOI:** 10.3389/fnut.2025.1550142

**Published:** 2025-05-14

**Authors:** Melekşen Akin, Sadiye Peral Eyduran, Jelena Mileševic, Suzana Pavlovic, Amil Orahovac, Marta W. Vasconcelos, Marija Knez

**Affiliations:** ^1^Department of Horticulture, Iğdır University, Iğdır, Türkiye; ^2^Department of Horticulture, Muğla Sıtkı Koçman University, Fethiye, Türkiye; ^3^Centre of Research Excellence in Nutrition and Metabolism, Institute for Medical Research, National Institute of Republic of Serbia, University of Belgrade, Belgrade, Serbia; ^4^CAPNUTRA Capacity Development in Nutrition, Belgrade, Serbia; ^5^Faculty of Food Safety, Food Technology and Ecology, University of Donja Gorica, Podgorica, Montenegro; ^6^Laboratório Associado, Escola Superior de Biotecnologia, CBQF-Centro de Biotecnologia e Química Fina, Universidade Católica Portuguesa, Porto, Portugal

**Keywords:** analysis, network analysis, performance analysis, *Pisum sativum*, science mapping

## Abstract

Pea (*Pisum sativum* L.) is a nutritious legume with health benefits, gaining attention as a functional food. Bibliometric studies use quantitative methods to assess research trends, gaps, and future directions. The main objective of this study was to provide a comprehensive overview of the fragmented literature on the nutritional profiles and health benefits of peas using a bibliometric approach. The analysis examined publications from 2013 to 2023, revealing trends in publication volume, author productivity, and international collaboration. Publications peaked in 2015, focusing on topics such as dietary fibers, carotenoids, phenolic compounds, and antinutrients affecting mineral bioavailability. Over the decade, the annual growth rate was 3.25%. The University of Saskatchewan produced the most influential research, with Warkentin TD as the most productive author. Canada and Poland had the highest number of publications, with the USA, China, and India following. Six major international co-authorship networks were identified, highlighting significant collaborations between countries. Key research themes included antioxidants, protein, fiber, and phytate in peas. This study provides a strong foundation for future integrated research, helping to better understand the potential of peas as a functional food and guiding more targeted studies to address current knowledge gaps across various disciplines.

## Introduction

Peas (*Pisum sativum* L.) are among the oldest and most extensively cultivated crops in the world. They are a rich source of protein and are consumed in both their green and dried forms (1, 2). They are also processed into various products, including frozen and canned forms, offering versatility in different culinary applications. Peas possess a high nutritional profile and offer various potential health benefits, including anti-cancer, anti-obesity, and cardio-protective effects (3). These health-promoting properties are attributed to their rich content of functional compounds, such as proteins, minerals, vitamins, fatty acids, and carbohydrates. The outer pod, which constitutes around 40% of the peas’ fresh weight, is typically discarded. However, this unused material can either be repurposed as animal feed or result in waste and biomass loss (4).

Over the years, the phytochemical profile of peas has gained attention due to their antioxidant activity and health-promoting effects. Phenolic compounds are among the most well-known phytochemicals that may help protect against chronic illnesses, including inflammatory-related disorders and cancers (5). Peas contains various phenolic compounds, including flavonoids such as kaempferol and quercetin derivatives, as well as phenolic acids like ferulic, p-coumaric, and gallic acid (6). Condensed tannins are also present, influencing both nutritional and sensory properties. Additionally, lignans and other polyphenols contribute to its antioxidant potential (7). Consuming peas over long term may offer protection against the development of diabetes, cancer, cardiovascular diseases, and degenerative illnesses, which can be attributed to their rich polyphenol content (8). In addition, peas are also high in dietary fibers, carbohydrates, amino acids, proteins, and minerals, containing high-quality fatty acids that contribute to the prevention of conditions such as arthritis, inflammation, hypertension, and coronary heart disease (3). Peas are naturally gluten-free, making them a great option for individuals with celiac disease.

Although peas contain a wide range of beneficial components, they also have certain anti-nutritional factors (also called non-nutrients), such as phytic acid, lectins, and trypsin inhibitors, which can interfere with nutrient absorption. Despite these antinutrients can reduce nutrient bioavailability, several recent studies have also highlighted their role as bioactive compounds due to their metabolic and physiological benefits (9–11). Phytic acid forms insoluble complexes with minerals like copper, iron, and zinc, reducing their bioavailability in the human gastrointestinal tract (12). While iron biofortification in staple foods has been promoted as a cost-effective strategy to reduce global iron deficiency, it has been shown that simply increasing iron content in biofortified crops may not be sufficient to significantly improve iron status in iron-deficient populations (13). A more effective approach involves plant breeding to produce low-phytate crops with higher iron bioavailability, as these crops can offer up to 2.5 times greater iron absorption (14). Processing methods such as soaking, roasting, boiling, pressure cooking, and sprouting can also be effective in reducing phytic acid content, thus improving mineral absorption (15). Reducing anti-nutritional factors like phytates in peas enhances the bioavailability of key nutrients such as iron, zinc, and calcium (16). Developing low-phytate pea varieties can greatly enhance nutrient absorption, making them a key component in strategies to combat multiple micronutrient deficiencies, particularly when paired with other nutrient-rich foods. However, the substantial presence of polyphenolic compounds in peas still poses a challenge by inhibiting iron absorption. Studies by Liu et al. (17) demonstrated that pea varieties with unpigmented seed coats containing lower levels of polyphenols had seven times higher iron bioavailability than those with pigmented coats. Although peas are not a staple food, they can reduce micronutrient deficiencies when included in a balanced diet. Ongoing research into selective breeding and genetic modification aims to improve the nutritional profile of peas further, increasing their contribution to better health outcomes. With the rising incorporation of pea-derived ingredients in various food products, there is also an increasing concern regarding pea-related allergens. Among the most notable allergenic proteins identified are Pis s 1, Pis s 2, and albumins PA1 and PA2, alongside a non-specific lipid transfer protein (18). Pea allergens may show cross-reactivity with other legumes, including lentils and peanuts, due to structural similarities in their protein compositions. This cross-reactivity is particularly observed with vicilin proteins, which are prevalent allergens in both peas and lentils (19). The impact of food processing on the allergenic properties of peas remains incompletely understood. Some evidence indicates that blanching may decrease allergenicity for certain individuals, but further investigation is required to validate these observations and examine additional processing techniques (18). As plant-based dietary patterns increasingly become prevalent, it is important to comprehend and mitigate the allergenic risks associated with peas to guarantee food safety and protect consumer health.

Bibliometric analysis is a research method used to systematically evaluate and quantify trends, patterns, and key topics within a specific scientific field. It involves the use of quantitative techniques to analyze published documents across various scientific journals, allowing for the identification of research growth, emerging themes, and influential studies (20). By conducting bibliometric analyses, researchers can gain a deeper understanding of the evolution of a particular field, including shifts in focus and areas that may require further exploration. This approach is particularly useful for uncovering knowledge gaps, guiding future research directions, and highlighting under-explored topics that may offer valuable insights (21). Furthermore, bibliometric analysis serves as a tool for researchers, policymakers, and practitioners to stay informed about the current state of the field and make informed decisions about research priorities, funding allocations, and practical applications (22). Additionally, this assessment can help stakeholders identify the most significant research and partnerships that are influencing the field, thus encouraging evidence-based decision-making. It also provides a more thorough understanding of the connection between pea research and wider nutritional and health trends, thereby promoting innovation in food product development and public health initiatives. For the general public, these insights can increase awareness of the nutritional advantages of peas, thereby encouraging healthier eating habits and a greater incorporation of this nutrient-rich legume into daily diets (23).

The objective of this work is to explore and gain a deeper understanding of research trends and recommendations concerning the nutritional profile and health benefits of peas, as presented in publications from the past 10 years, using descriptive and retrospective bibliometric analysis techniques. This study also identifies trending topics and research gaps within the field, highlighting areas that require further investigation. Bibliographic data were retrieved from the Scopus database to address the following questions:

*RQ1*: *What is the distribution of publications related to the nutritional profile and health benefits of peas?* *RQ2*: *Which journals, authors, and institutions are most influential in the field of research on the nutritional profile and health benefits of peas, and what collaborative networks exist among researchers?* *RQ3*: *Which countries are leading research on the nutritional profile and health benefits of peas, and what are the underlying collaboration patterns among them?* *RQ4*: *What are the major research keywords used and the possible future research directions on nutritional profile and health benefits of peas?*

### Materials and methods

A bibliometric analysis was used to perform a systematic literature review. Systematic review relies on qualitative assessment and therefore it is open to interpretation bias. However, bibliometric analysis relies on quantitative methods and, thus can prevent interpretation bias (24). Consequently, the bibliometric approach allows academicians to explore vast volumes of data in comparison to systematic literature review in a transparent and replicable way (25). This study used bibliometrics to project the hot topics and research gaps in the field of nutritional composition and health benefits of peas. Performance analysis and science mapping techniques were implemented using Bibliometrix package (26) in R language (27).

The first step consisted of the bibliographic data extraction, using Scopus (Elsevier) core collection for the literature retrieval (28). The Boolean operators (AND and OR) and wildcards were used to detect documents with different combinations of the selected keywords including singular and plural forms. The following keyword combination was used to search within the titles, abstracts, and keywords of documents in the Scopus database:

“*Pisum sativum*” AND (“functional food*” OR nutraceutic* OR vitamin* OR antioxidant* OR polyphenol* OR phenol* OR flavonoid* OR flavan* OR anthocyanin* OR metabolomic* OR phytochemical* OR “organic acid*” OR “secondary metabolites” OR bioactivity OR “bioactive compound*” OR Carbohydrate* OR fiber* OR protein* OR “amino acid*” OR “fatty acid” OR mineral* OR macroelement* OR microelement* OR macronutrient* OR micronutrient* OR vitamin* OR *toxin* OR anti-nutrient* OR antinutrient* OR “protease inhibitor*” OR phytate* OR “phytic acid” OR oxalate* OR lectin* OR tannin* OR saponin* OR amylase* OR oligosaccharide* OR trypsin*) AND (health OR pharmacol* OR diet* OR nutrition*) AND NOT (“grass pea*” OR “butterfly pea” OR “zombi pea” OR fish* OR aquaculture)

**FIGURE 1:**
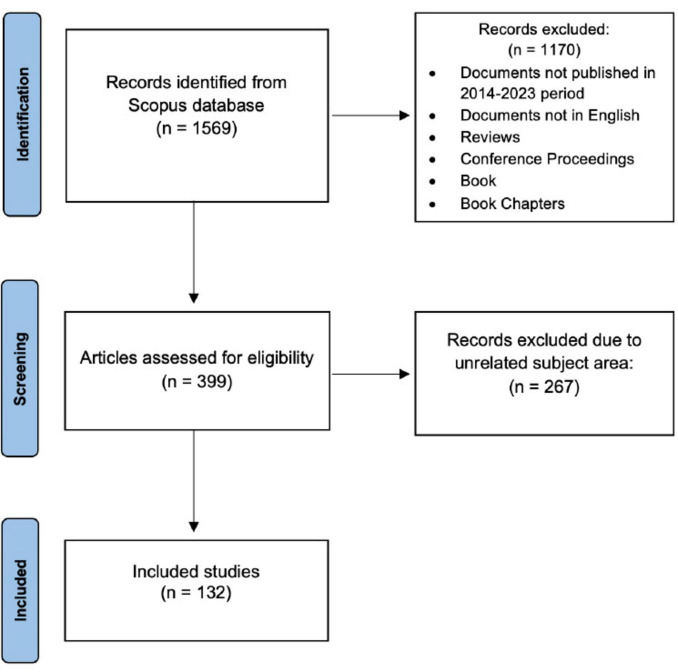
PRISMA diagram showing the different steps of bibliographic data identification.

The search was conducted in June 2024, and the syntax returned 1569 publications in the Scopus database. To refine the dataset, we applied several filters based on predefined inclusion and exclusion criteria. The inclusion criteria encompassed publications from the period 2013-2023 to ensure the relevance of recent research, documents written in English to maintain consistency and accessibility, and only peer-reviewed research articles to ensure scientific rigor. The exclusion criteria involved removing book chapters, conference proceedings, data papers, and notes, as these sources often lack the depth, peer-review process, or original research focus required for a comprehensive bibliometric analysis. These filtering steps reduced the dataset to 399 records before the full-text review. A subsequent full-text review was conducted to exclude publications that, despite matching the search terms, were not directly relevant to the research topic. The final dataset was reduced to 132 papers following the filtering process. The entire screening procedure is illustrated in Figure 1, according to PRISMA guidelines (29).

The bibliographic data was examined with bibliometric analysis using performance analysis and science mapping techniques (30). Performance analysis highlights the overall trend of the field including the number of documents or citations of the publications within the bibliographic data and compiles them by author, journal, country, and institution (28). The h-index is the most popular metric evaluating the scientific impact of researchers or journals which refers to the publication number that have received at least h citations (31). Although the h-index is considered to be a robust indicator to examine impact of scientific productivity (32), it is not a suitable metric when authors from different scientific areas or authors with different seniority stages are to be compared (33). The m-index which is the h-index divided by the number of years that have passed between an author’s first and latest publication helps to overcome the problems with comparing researchers at various levels of their career (34). The science mapping technique projects the hidden patterns in the conceptual, social, and intellectual structure of a particular scientific field and its dynamic evolution over time (35). The conceptual structure shows the connections that can emerge among various concepts or keywords. The social structure delineates the relationships that can appear between different units of analysis including authors, affiliations, and countries. The intellectual structure refers to the associations among different nodes (such as documents, journals, authors) that can demonstrate evolutions in a given field. The most used techniques to conduct these kinds of analyses are co-occurrence analysis and co-citation analysis (28). A co-occurrence analysis was performed using author keywords to capture the conceptual structures related to nutritional composition and health benefits of peas. In this regard, the association strength normalization (36) and the Louvain cluster algorithm (37) were adopted resulting in 50 nodes. A co-authorship analysis was conducted based on co-authored documents (38) to capture the social structure, in which 50 authors consisted the unit of analysis adopting the association strength normalization and the Louvain cluster algorithm (28, 37). Isolated nodes were not discarded to show a more comprehensive overview of the degree of collaboration existing between researchers in this field.

## Results and discussion

### Research question 1

**FIGURE 2:**
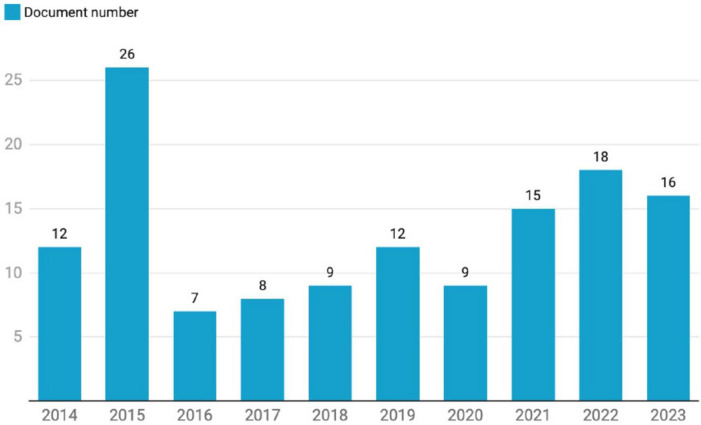
Scientific publication progress between 2014 and 2023 years (3.25% annual growth rate). The y-axis maps publication number and x-axis maps year.

This question aimed to examine the literature on peas’s nutritional profile and health benefits. The first finding addresses the initial research question regarding the distribution of articles published between 2014 and 2023 on this topic. As illustrated in Figure 2, the number of publications varied over the years, peaking in 2015 with 26 documents. The papers published in 2015 focused on various aspects of pea research, including bean seed fibers’ structural and functional properties (17, 39, 40). Studies also explored the content of carotenoids (41), phenolic compounds, and antioxidant activity (42–44). Additionally, some research that year examined antinutrient content and its impact on mineral bioavailability, particularly iron (13, 17, 45–47). Over the past decade, the annual growth rate of publications in this field was 3.25%.

### Research question 2

**FIGURE 3:**
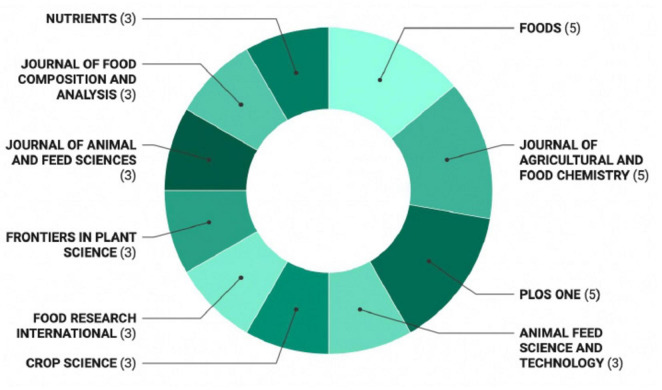
Most prolific journals in the area of nutritional composition and health benefits of peas based on total publication number (TP).

The second research question aimed to identify the most prolific journals, authors, and institutions in the field of peas’ nutritional profile and health benefits, as well as uncover hidden collaboration networks among authors. Figure 3 presents the journals with the highest number of publications on this topic based on total publications (TP). The most relevant journals in nutritional profile and health benefits of peas research, each with five publications, were Foods, Journal of Agricultural and Food Chemistry, and PLoS ONE. Other active journals, each with three publications, included Crop Science, Food Research International, Frontiers in Plant Science, Journal of Animal and Feed Sciences, Journal of Food Composition and Analysis, and Nutrients.

**FIGURE 4:**
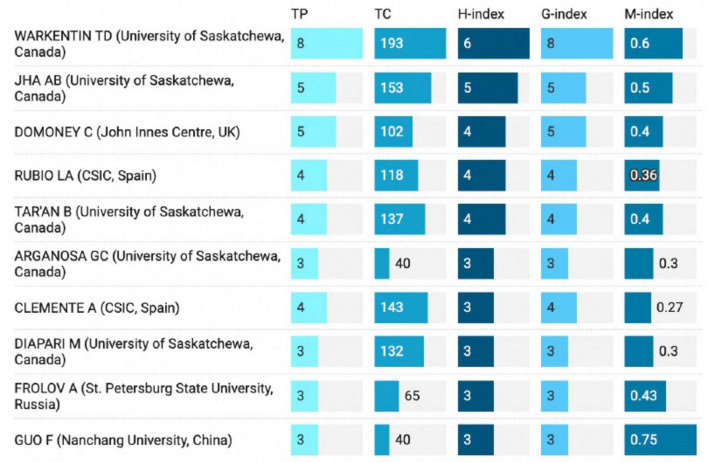
Graphical representation of the most active authors in the field of nutritional composition and health benefits of peas based on total publication number (TP). Author total citation (TC), H-index, G-index, and M-index are also shown.

Research question two also identified the most active authors working on the nutritional profile and the determination of the health benefits of peas. The most productive authors on the nutritional profile and health benefits of peas in terms of TP, H-index, G-index, and M-index are presented in Figure 4. According to our data collection, the most productive author was Warkentin TD from the University of Saskatchewan, Canada, with eight publications, 193 citations, an H-index of 6, a G-index of 8, and M-index of 0,6. The second most active author was JHA Jha AB, from University of Saskatchewan, Canada, with a total of 5 publications, 153 citations, an H-index of 5, G-index of 5, and M-index of 0.5, followed by Domoney C from John Innes Centre, United Kingdom, with a total of 5 publications, 102 citations, an H-index of 4, G-index of 5, and M-index of 0.4. Most top authors in the field were from the University of Saskatchewa, Canada.

Warkentin TD investigated the composition and protein quality of peas (48), as well as the bioavailability of iron (14, 17). In collaboration with Jha AB, he studied protein digestibility (49), SNP variations, and crude protein concentration (50). Pea protein, a widely used ingredient in meat alternatives, is rich in lysine but relatively low in tryptophan and sulfur-containing amino acids such as methionine and cysteine (51). Research linking genetic loci to the nutritional properties of peas is essential, as variability in their nutritional composition can result from factors like genetic background and environmental conditions (48). Zhou et al. (49) identified three loci associated with methionine and cysteine concentrations, four linked to tryptophan, three to lysine, and two related to the *in vitro* protein digestibility. Warkentin TD and Jha AB also examined the polyphenolic profile (52), carotenoid content (41), and folate content (50) of pea seeds, which serve as natural sources of antioxidants in food.

Domoney C and colleagues investigated the relationship between specific mutations and protein accumulation in pea seeds (53). They also explored starch assembly and its role in supporting healthy glucose homeostasis in humans (54). One strategy to promote healthy glucose levels is to increase the amount of resistant starch in the diet, which is fermentable by the colonic microbiota. Human studies have demonstrated that postprandial blood glucose levels and insulin sensitivity improve after supplementation with resistant starch, including in patients with type 2 diabetes (54). Additionally, Domoney C examined seeds’ quality and physicochemical properties (55) and the removal of anti-nutritional proteins from pea seeds (45). The presence of antinutritional factors, such as phytic acid, tannins, and protease inhibitors, significantly restricts the use of grain legumes as a food source (56). Trypsin inhibitors, in particular, reduce the digestion and absorption of dietary proteins by inhibiting the activity of pancreatic enzymes like trypsin and chymotrypsin, which can lower the overall nutritional value of legume-based foods (57). This is especially relevant for grass pea a highly resilient and protein-rich legume that holds great potential for food security and sustainable agriculture (58). However, the presence of antinutritional compounds, including protease inhibitors, has been a major challenge limiting its broader adoption in human diets (56). The study by Clemente et al. (45) highlights the importance of genetic mutations that reduce or eliminate the function of trypsin inhibitors, such as Bowman-Birk inhibitors, to improve seed quality. Such research is crucial for grass pea breeding programs, as it provides insights into strategies for enhancing its nutritional profile while maintaining its adaptability to harsh growing conditions. By addressing these antinutritional constraints, grass pea can become a more viable and widely accepted protein source for both human consumption and animal feed, contributing to diversified and sustainable food systems. Graphical representation of the most prolific authors in the field of nutritional profile and health benefits of peas research is provided in Figure 4.

A list of the most cited articles in the current bibliographic dataset, ordered by total citation number (TC), is also provided in Supplement material 1. The most cited paper in our data collection was “Structural and functional characteristics of dietary fiber in beans, lentils, peas and chickpeas, which examined the dietary fiber properties of peas and beans (40). The second most cited study was “Characterization of pea (*Pisum sativum*) seed protein fractions,” which focused on the characterization of pea protein (59).

**FIGURE 5:**
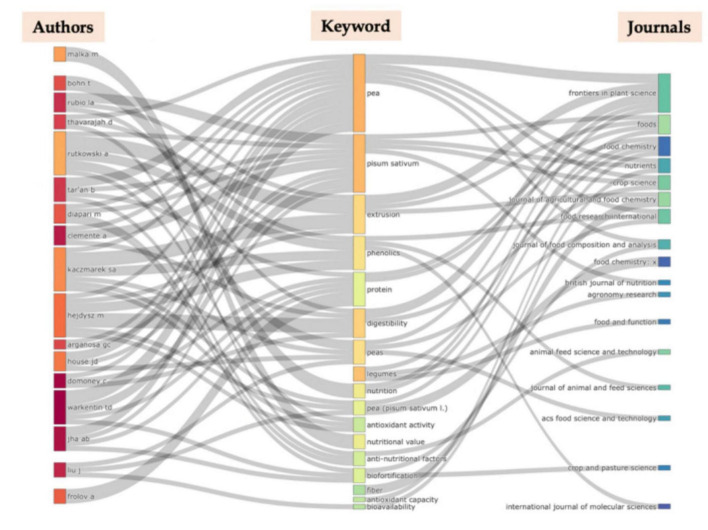
Sankey plot demonstrates the associations among top authors, most frequent keywords and top journals in the field of nutritional composition and health benefits of pea.

Figure 5 projects the association between top authors, keywords, and journals in the field of nutritional profile and health benefits of peas. A bibliometric analysis revealed the interconnections between key contributors, relevant keywords, and leading journals that have shaped research in this area. These associations highlight prominent themes, collaboration networks, and research trends within the field.

Malka M was associated with the keyword “nutritional value,” while Bohn T focused on “nutrition,” with publications in Frontiers in Plant Science and Food. Rubio LA was linked to the keyword “digestibility,” with research published in Frontiers in Plant Science and Crop Science. Thavarajan D worked on “phenolics” and “anti-nutritional factors,” while Jha AB explored “biofortification,” with work published in Crop and Pasture Science. The remaining associations between top authors, keywords, and journals are presented in Figure 5.

**FIGURE 6:**
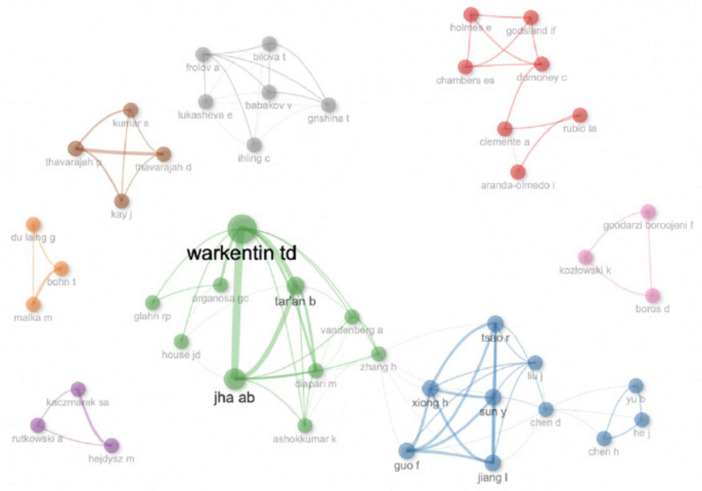
Collaboration networks among top authors in the area of nutritional composition and health benefits of peas based on > 1 collaborative paper.

Figure 6 illustrates the hidden collaboration networks among the top authors in the nutritional profile field and peas’ health benefits, based on at least one collaborative paper. Eight distinct subnetworks were identified among the most prolific authors. Warkentin D emerged as the most actively collaborating author within the green cluster, working closely with Jha AB, Tar’an B, and others. Another large collaboration group was between Jiang L, Tsao R, Guo F, etc.

Rubio LA collaborated with Clemente A, Domoney C, and others, forming a distinct subnetwork. Frolov A, on the other hand, formed a separate subnetwork with Babakov V, Lukasheva E, and colleagues. The entire map of collaboration groups is presented in Figure 5.

### Research question 3

**FIGURE 7:**
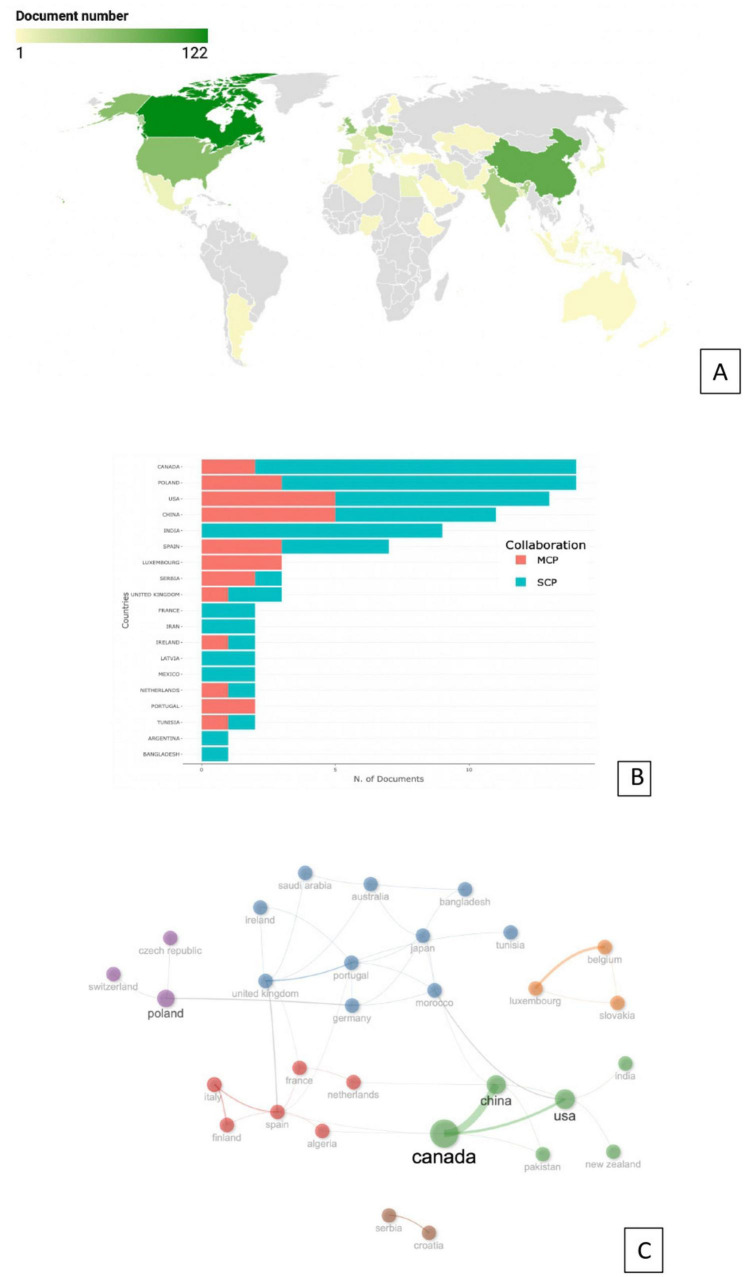
Graphical representation of most productive countries in nutritional composition and health benefits of peas based on total publication number: **(A)** world map. **(B)** International collaboration. SCP: single country publication, MCP: multiple country publication. **(C)** Collaboration network based on > 1 collaborative paper.

The third research question aimed to identify the most prolific countries and uncover the hidden collaboration networks among them in the field of nutritional profile and health benefits of peas. Figure 7A visualizes the most significant countries based on publication numbers. Country Scientific Production measures the number of authors’ appearances by country affiliations, which means that if in an article 5 authors are working in Canada, this article will be counted 5 times for this country. Therefore, the sum of the production indicator can exceed the total number of articles. Figure 7B projects the corresponding author’s country for each document, associating it with a single country based on the corresponding author’s affiliation. This helps identify the geographical distribution of research in the field of nutritional profile and health benefits of peas. The frequency per country in this map shows the total number of publications.

Additionally, this analysis measures the proportion of records in which at least one author is affiliated with a country different from that of the corresponding author. The index is labeled as Multiple Country Publications (MCP) (26). As presented in Figure 7A, the most productive countries in the field of nutritional profile and health benefits of peas were Canada (122 documents), China (88 documents), USA (66 documents), UK (61 documents), Poland (52 documents), etc. The bibliometric analysis results indicate that Canada and Poland had the same number of documents based on corresponding author affiliations (Figure 7B). Furthermore, most of the articles were single-country publications, highlighting a trend of national-focused research in this field. The USA was ranked third, followed by China in fourth place. India secured fifth place, with no international collaboration in the papers where the corresponding author was from India. Spain ranked sixth, with an almost equal number of global and single-country publications. Notably, all publications from Luxembourg involved collaboration with other countries (Figure 7B).

Next, based on at least one collaborative paper, this study examined the relationship of co-authorship between countries in the field of nutritional profile and health benefits of peas. Six distinct collaboration subnetworks were identified among countries, highlighting global cooperation in this research area (Figure 7C). The largest international collaboration network was between UK, Ireland, Germany, Morocco, Japan, Bangladesh, Australia, Portugal, Tunisia, and Saudi Arabia. Another collaboration network was between Canada, China, USA, New Zealand, Pakistan, and India, with Canada being the most active within the group. Serbia and Croatia formed the smallest collaboration network, while Belgium, Luxembourg, and Slovakia constituted a separate collaboration group. Poland, Switzerland, and the Czech Republic formed another distinctive sub-network. Additionally, Italy, Finland, Spain, Algeria, France, and the Netherlands were grouped in a separate collaboration network (Figure 7C).

### Research question 4

**FIGURE 8:**
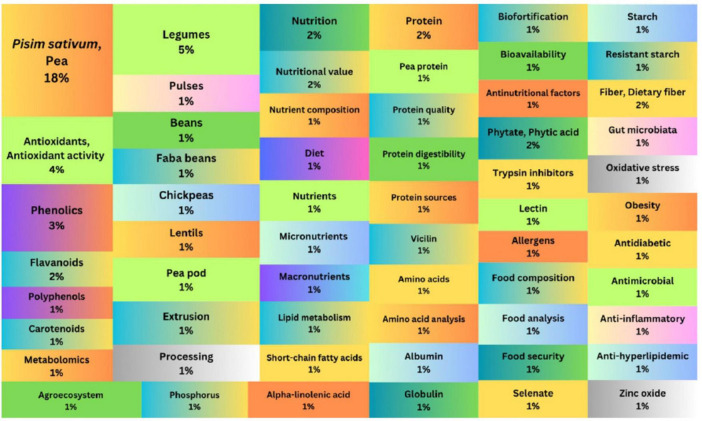
Tree-map displaying the keyword occurrence percentages in the field of nutritional composition and health benefits of peas.

The fourth research question aimed to pinpoint the most frequently used author keywords in the field of nutritional profile and health benefits of peas. Figure 8 maps the keywords with the highest occurrence. The occurrence percentages of the most frequently used keywords were as follows: Pea and *Pisum sativum* (18%), Legumes (5%), Antioxidants and Antioxidant activity (4%), Phenolics (3%), Flavonoids (2%), Nutrition (2%), Nutritional value (2%), Protein (2%), Phytate and Phytic acid (2%), Fiber and dietary fiber (2%), Polyphenols (1%), Carotenoids (1%), Metabolomics (1%), Pulses (1%), Beans (1%), Faba beans (1%), Chickpeas (1%), Lentils (1%), Pea pod (1%), Extrusion (1%), Processing (1%), Nutrient composition (1%), Diet (1%), Nutrients (1%), Macronutrients (1%), Micronutrients (1%), Lipid metabolism (1%), Short-chain fatty acids (1%), Pea protein (1%), Protein quality (1%), Protein digestibility (1%), Protein sources (1%), Vicilin (1%), Amino acids (1%), Amino acid analysis (1%), Albumin (1%), Globulin (1%), Agroecosystem (1%), Biofortification (1%), Bioavailability (1%), Anti-nutritional factors (1%), Trypsin inhibitors, Lectin (1%), Allergens (1%), Food composition (1%), Food analysis (1%), Food security (1%), Starch (1%), Resistant starch (1%), Gut microbiota (1%), Oxidative stress (1%), Obesity (1%), Antidiabetic (1%), Antimicrobial (1%), Anti-inflammatory, etc.

These primary research keywords capture the trending topics in peas’ nutritional profile and health benefits, illuminate literature gaps, and suggest potential directions for future research. Analyzing the most frequently used keywords reveals significant research gaps, particularly in underexplored areas such as biofortification strategies, the effects of anti-nutritional factors, and their implications for human health. Future research could bridge these gaps by delving deeper into the genetic mechanisms responsible for nutrient variability in peas, which could help develop varieties with enhanced nutritional profiles. Investigating ways to improve the bioavailability of essential minerals, such as iron, zinc, and calcium, through selective breeding or genetic modification will be vital in making peas even more nutritionally beneficial. Additionally, further exploration of the role of peas in managing chronic diseases, such as diabetes, cardiovascular disorders, and inflammatory conditions, could uncover their potential as a functional food. Studies could also focus on how bioactive compounds in peas, such as phenolic compounds and dietary fiber, contribute to disease prevention and overall health improvement, providing a more holistic understanding of peas as part of a health-promoting diet. Finally, more international collaboration could enhance the exchange of knowledge and foster innovative approaches to optimize the nutritional potential of peas.

The answers of these questions can assist the research community in finding where to publish their work and with whom they can collaborate with, as well as what are the trending research topics and what might be the potential future research directions on the topic of nutritional profile and health benefits of pea. This study underscores significant trends, key research, and knowledge gaps that will direct future studies and encourage teamwork, aiding in the prioritization of research initiatives aimed at addressing new health issues and maximizing the nutritional benefits of peas. These answers can help nutritionists in staying informed about the most recent scientific findings, enabling them to create evidence-based dietary guidelines and gain a deeper insight into the health benefits of peas. For the broader community, a bibliometric study on the health advantages of peas can underscore scientifically validated reasons for their dietary inclusion, such as enhancing gut health, benefiting heart wellness, stabilizing blood sugar levels, and supplying vital nutrients like protein, fiber, and micronutrients. Additionally, it can clear up prevalent misunderstandings regarding legumes, fostering a deeper appreciation of their nutritional significance and functional attributes.

One of the limitations of this study is the restricted access to information, as it solely relied on the Scopus database for identifying documents to undergo bibliometric analysis. Other databases, such as Springer Link, PubMed, or Web of Science, may have yielded different results and provided additional insights. Furthermore, the scope of the findings could have been more focused depending on the specific keyword combinations used in the search. The depth of the analysis could have also been enhanced if a broader range of keywords or keyword combinations related to the nutritional composition and health benefits of peas had been incorporated. Despite the limitation of using only the Scopus database, its comprehensive coverage of peer-reviewed journals, conference proceedings, and research articles provided a solid foundation for the bibliometric analysis. Scopus is widely recognized for its broad inclusion of high-quality scientific literature, ensuring that the most relevant and impactful studies in the field of nutritional profile and health benefits of peas were captured. Focusing on a specific database also allowed for a more streamlined and manageable dataset, minimizing the potential for information overload and making the analysis more targeted. While other databases may offer different perspectives, Scopus’ robustness and consistency provided a reliable and well-rounded view of the research landscape. The selected keywords were thoughtfully chosen to encompass most studies related to the nutritional composition and health benefits of peas, allowing for a focused and relevant exploration of the topic. While broadening the range of keywords could provide additional insights, it may also risk diluting the core findings. Furthermore, although some less significant keywords may have been omitted, the studies included in our review are likely to cover the essential concepts, ensuring that the most pertinent aspects of peas’ nutritional composition and health benefits are thoroughly addressed.

## Conclusion

Peas are a widely cultivated pulse with a rich nutritional profile, offering health benefits through bioactive compounds like polyphenols, though antinutritional factors can limit nutrient absorption. Despite these challenges, peas are a versatile, gluten-free functional food. Given the fragmented literature on their nutritional properties, a bibliometric analysis is essential to consolidate knowledge and guide future research aimed at maximizing their potential in food applications and public health. Performed bibliometric analysis revealed key trends in pea research from 2013 to 2023. The number of publications fluctuated over the years, peaking in 2015 with 26 papers, which focused on topics such as fiber properties, carotenoid and phenolic content, antioxidant activity, and the impact of antinutrients on mineral bioavailability. Over the decade, the annual growth rate of publications was 3.25%. The most productive author was Warkentin TD from the University of Saskatchewan, Canada, followed by Jha AB and Domoney C. The most cited paper examined dietary fiber properties in legumes, while another highly cited study focused on pea protein characterization. Canada and Poland had the highest number of publications based on corresponding author affiliations, with the USA, China, and India following. Most studies were single-country publications, with Luxembourg being an exception, as all its papers involved international collaboration. Six major co-authorship networks were identified, with the largest involving the UK, Ireland, Germany, and other countries, while Canada played a central role in another group with the USA, China, and India. This bibliometric analysis provides valuable insights into the current state of pea research, highlighting key trends, identifying knowledge gaps, and offering a foundation for future studies to optimize the health benefits and applications of peas.

## Data Availability

The raw data supporting the conclusions of this article will be made available by the authors, without undue reservation.
