# Supplementary material for: Nutritional composition and health benefits of peas—a bibliometric research
Source: Front Nutr. 2025 May 14;12:1550142. doi: 10.3389/fnut.2025.1550142 (PMC12116326; doi:10.3389/fnut.2025.1550142)
Supplement: Supplementary file 1 [file Table_1.docx]

**Supplement 1.** Top publications with highest citation in the Scopus database ordered by total citation number (TC).

| # | Document | TC | TC/Year |
| --- | --- | --- | --- |
| 1 | Brummer, Y., Kaviani, M., & Tosh, S. M. (2015). Structural and functional characteristics of dietary fibre in beans, lentils, peas and chickpeas. ***Food Research International***, *67*, 117-125. | 148 | 14.80 |
|  |  |  |  |
|  |  |  |  |
| 2 | Rubio, L. A., Perez, A., Ruiz, R., Guzmán, M. Á., Aranda‐Olmedo, I., & Clemente, A. (2014). Characterization of pea (Pisum sativum) seed protein fractions. ***Journal of the Science of Food and Agriculture***, *94*(2), 280-287. | 96 | 8.73 |
|  |  |  |  |
|  |  |  |  |
| 3 | Salmean, Y. A., Segal, M. S., Palii, S. P., & Dahl, W. J. (2015). Fiber supplementation lowers plasma p-cresol in chronic kidney disease patients. ***Journal of Renal Nutrition***, *25*(3), 316-320. | 92 | 9.20 |
|  |  |  |  |
|  |  |  |  |
| 4 | Jha, A. B., Ashokkumar, K., Diapari, M., Ambrose, S. J., Zhang, H., Tar’an, B., ... & Purves, R. W. (2015). Genetic diversity of folate profiles in seeds of common bean, lentil, chickpea and pea. ***Journal of food composition and analysis***, *42*, 134-140. | 67 | 6.70 |
|  |  |  |  |
|  |  |  |  |
| 5 | Nosworthy, M. G., Franczyk, A. J., Medina, G., Neufeld, J., Appah, P., Utioh, A., ... & House, J. D. (2017). Effect of processing on the in vitro and in vivo protein quality of yellow and green split peas (Pisum sativum). ***Journal of Agricultural and Food Chemistry***, *65*(35), 7790-7796. | 66 | 8.25 |
|  |  |  |  |
|  |  |  |  |
| 6 | Chen, H., Mao, X. B., Che, L. Q., Yu, B., He, J., Yu, J., ... & Chen, D. W. (2014). Impact of fiber types on gut microbiota, gut environment and gut function in fattening pigs. ***Animal feed science and technology***, *195*, 101-111. | 63 | 5.73 |
|  |  |  |  |
|  |  |  |  |
| 7 | Saget, S., Costa, M., Santos, C. S., Vasconcelos, M. W., Gibbons, J., Styles, D., & Williams, M. (2021). Substitution of beef with pea protein reduces the environmental footprint of meat balls whilst supporting health and climate stabilisation goals. ***Journal of Cleaner Production***, *297*, 126447. | 51 | 12.75 |
|  |  |  |  |
|  |  |  |  |
|  |  |  |  |
| 8 | Świeca, M., & Gawlik-Dziki, U. (2015). Effects of sprouting and postharvest storage under cool temperature conditions on starch content and antioxidant capacity of green pea, lentil and young mung bean sprouts. ***Food chemistry***, *185*, 99-105. | 50 | 5.00 |
|  |  |  |  |
|  |  |  |  |
| 9 | Jakubczyk, A., & Baraniak, B. (2014). Angiotensin I converting enzyme inhibitory peptides obtained after in vitro hydrolysis of pea (Pisum sativum var. Bajka) globulins. ***BioMed Research International***, *2014*(1), 438459. | 50 | 4.55 |
| 10 | Ashokkumar, K., Diapari, M., Jha, A. B., Tar’an, B., Arganosa, G., & Warkentin, T. D. (2015). Genetic diversity of nutritionally important carotenoids in 94 pea and 121 chickpea accessions. ***Journal of Food Composition and Analysis***, *43*, 49-60. | 44 | 4.40 |
|  |  |  |  |
|  |  |  |  |
|  |  |  |  |
